# Antioxidant, antifungal, and aphicidal activity of the triterpenoids spinasterol and 22,23-dihydrospinasterol from leaves of *Citrullus colocynthis* L.

**DOI:** 10.1038/s41598-022-08999-z

**Published:** 2022-03-22

**Authors:** Maqsood Ahmed, Allah Rakha Sajid, Ansar Javeed, Muhammad Aslam, Taswar Ahsan, Dilbar Hussain, Abdul Mateen, Xiuwei Li, Peiwen Qin, Mingshan Ji

**Affiliations:** 1 College of Plant Protection, Shenyang Agricultural University, Shenyang, 110866 People’s Republic of China; 2 Department of Agriculture (Plant Protection) Pest Warning & Quality Control of Pesticides, Gujrat, 50700 Pakistan; 3 Department of Agriculture (Plant Protection) Pest Warning and Quality Control of Pesticides, Lahore, 54800 Pakistan; 4 School of Life Sciences, Henan University, Jinming Campus, Kaifeng, Henan China; 5 Department of Resources and Environmental Microbiology, College of Land and Environment, Shenyang Agricultural University, Shenyang, 110866 People’s Republic of China; 6 Entomological Research Institute, Ayub Agriculture Research, AARI, Faisalabad, 38070 Pakistan; 7 College of Plant Protection, China Agricultural University, Beijing, 100193 China

**Keywords:** Biochemistry, Plant sciences

## Abstract

Terpenoids from natural plant sources are valuable for their diverse biological activities that have important roles in the medical and agrochemical industries. In this study, we assessed the antioxidant, antifungal, and aphicidal activities of a mixture of spinasterol and 22,23-dihydrospinasterol from the leaves of *Citrullus colocynthis*. We used 1,1-diphenyl-2-picrylhydrazyl (DPPH) to assess antioxidant activity, and we measured antifungal activity using mycelium growth inhibition assays with three pathogenic fungi, *Magnaporthe grisea*, *Rhizoctonia solani*, and *Phytophthora infestans.* Aphicidal activity against adults of *Myzus persicae* was determined using in vitro and in vivo assays. Spinasterol and 22,23-dihydrospinasterol exhibited moderate antioxidant activity, even at lower concentrations: 19.98% at 0.78 µg mL^−1^, 31.52% at 3.0 µg mL^−1^, 36.61% at 12.5 µg mL^−1^, and 49.76% at 50 µg mL^−1^. Spinasterol and 22,23-dihydrospinasterol showed reasonable levels of fungicidal activity toward *R. solani* and *M. grisea*, with EC_50_ values of 129.5 and 206.1 µg mL^−1^*,* respectively. The positive controls boscalid and carbendazim were highly effective against all fungi except boscalid for *M. grisea* (EC_50_ = 868 µg mL^−1^) and carbendazim for *P. infestans* (EC_50_ = 8721 µg mL^−1^). Significant insecticidal activity was observed in both residual and greenhouse assays, with LC_50_ values of 42.46, 54.86, and 180.9 µg mL^−1^ and 32.71, 42.46, and 173.8 µg mL^−1^ at 72, 48, and 24 h, respectively. The antioxidant activity of spinasterol and 22,23-dihydrospinasterol was strongly positively correlated with their antifungal and insecticidal activity. Spinasterol and 22,23-dihydrospinasterol therefore show good antioxidant and aphicidal activity with moderate fungicidal activity, making them suitable candidates for an alternative to synthetic agents.

## Introduction

Oxidative stress plays a double role in infections; the pathologies that arise during such infections can be attributed to oxidative trauma and the creation of reactive species, often with lethal consequences. Microbial resistance to conventional antibiotics poses a significant threat to the treatment of infectious diseases. However, phytochemicals exhibit latent biological activity towards both resistant and sensitive pathogens. Phytochemicals are a valuable source of bioactive compounds with antimicrobial activities. Among these phytochemicals, phenolics are diverse secondary metabolites such as tannins, flavonoids, and lignin that exhibit antioxidant properties and are abundant in plant tissues. Likewise, reactive oxygen species (ROS) are produced as typical products in plant cellular breakdown. Naturally occurring compounds play a vital role against microbial resistance in the management of infectious diseases. Medicinal plants are prized sources of phytochemical compounds with biological activities and are used in the pharmacological and agrochemical industries. Synthetic chemicals are easily available and widely used as antioxidant, antimicrobial, antifungal, and pesticidal compounds, but their intensive, continuous use has caused the development of pest resistance and also has harmful effects on human and environmental health^1^.

*Citrullus colocynthi*s from the order Cucurbitales and the family Cucurbitaceae is an important plant for both medicinal and pesticidal purposes. *C. colocynthis* appears to exhibit anti-carcinogenic, antibacterial, antifungal, antidiabetic, and antioxidant properties and also shows insecticidal potential against various harmful insects^2–5^. Several biologically active compounds have been described from *C. colocynthis*; these include cucurbitacin E, I, J, K, and L^6^, cucurbitacin glycosides^7,8^ such as cucurbitacin glucoside I and L^8^, flavonoids, and flavone glycosides^8,9^. The insecticidal activity of *C. colocynthis* against numerous insect pests has also been evaluated^10^. In a recent study, the biologically active compounds spinasterol and 22,23-dihydrospinasterol were characterized from *C. colocynthis* leaves and evaluated against adult-stage *Brevicoryne brassicae* (Hemiptera: Aphididae), showing significant insecticidal properties^11^. Pronounced antioxidant activities were also reported from extracts of *C. colocynthis* leaves and roots^12^.

Spinasterol and 22,23-dihydrospinasterol are triterpenoids that are produced by a number of plants. Phytochemical analysis of *Bryony* *callus* Rattler leaves revealed that they contain β-sitosterol, triterpenes, spinasterol, 22,23-dihydrospinasterol, glycosides, and phenolics. An extract from *B. callus* was effective for the control of *Aedes aegypti* larvae, and larval mortality may have been attributed to the presence of phenolics, spinasterol, and 22,23-dihydrospinasterol. Larvicidal activity has also been reported for extracts from *Heliotropium indicum* and *Melothria maderaspatana*^13^. An extract from the leaves of *Mukia maderaspatana* showed potential antioxidant properties because of the presence of spinasterol, 22,23-dihydrospinasterol, flavonoids, and phenolics^14^. It scavenged 2,2′-azino-bis-3-ethylbenzothiazoline-6-sulfonic acid (ABTS) and DPPH radical molecules, which also possess reducing power^15^. A pharmacological study of *Bougainvillea spectabilis* stems reported that they contain caffeic acid, spinasterol, and 22,23-dihydrospinasterol and have been used in herbal medicines against cancer and hepatitis^16^. The leaves of *Vitex negundo* L., which contain salicylic acid and 22,23-dihydro-α-spinasterol-β-d-glucoside, repelled and were toxic to different strains of *Tribolium castaneum*^17^.

Two cucurbitane-type triterpenoid saponins were identified from a solvent extract of *C. colocynthis* fruit, but they were not assessed for antioxidant, antifungal, or insecticidal activities^18^. Similarly, a mixture of spinasterol and 22,23-dihydrospinasterol was isolated and characterized from roots of *Bermeuxia thibetica* (Lamiales: Lamiaceae), but it was not evaluated as an antimicrobial or insecticidal agent^19^. However, some biological activities of a triterpenoid spinasterol, 22,23-dihydrospinasterol, from *Melothria maderaspatana* (Cucurbitales: Cucurbitaceae) have been described^20^.

Green peach aphid (*Myzus persicae)* is a small green aphid that is the most significant pest of peach trees. It can harm more than 400 species of plants by feeding on plant sap; it causes decreased growth and shrinking of leaves that can lead to plant death. It is a vector of tobacco etch virus (TEV), cucumber mosaic virus (CMV), and potato virus Y (PVY), and it can also transmit various destructive viruses in other plants. Different synthetic pesticides are used to control this pest, including abamectin, cypermethrin, methylamine, and methylamine, which are the first agents for aphid control. However, continuous use of Imidacloprid or other pesticides may lead to the development of resistance^21^. Similarly, some botanical insecticides like *Azadirachta* (neem) and nicotine are also used to manage this pest^22^. In recent years, essential oils from plants have been used to control various pest; these include the essential oil from *Foeniculum vulgare*, a medicinal and culinary herb from the Mediterranean region^23,24^. Nonetheless, the use of such botanically based insecticides has been limited to date.

Although some research has been performed on the separation, purification, and characterization of biologically active compounds such as spinasterol and 22,23-dihydrospinasterol from natural plant sources, their isolation and identification from *C. colocynthis* and their use as antioxidant and antifungal agents has not yet been assessed. Here, guided by a detailed literature review and the known biological activities of these compounds, we evaluated a mixture of spinasterol and 22,23-dihydrospinasterol isolated from *C. colocynthis* for antioxidant activity and antifungal activity against *Magnaporthe grisea*, *Rhizoctonia solani*, and *Phytophthora infestans.* In a continuation of previous research, we also evaluated their activity against adults of *M. persicae*.

## Results

### Antioxidant activity

1,1-Diphenyl-2-picrylhydrazyl (DPPH) is a stable free radicle molecule in the form of a dark crystalline powder that is commonly used in laboratory research for antioxidant assays. It dissolves readily in acetonitrile and is recognized by its light absorption at 517 nm. It has violet color in solution and becomes colorless or pale yellow when neutralized by the scavenging activity of antioxidant molecules, causing a reduction in absorbance. Percent DPPH inhibition data were collected with various concentrations of a spinasterol and 22,23-dihydrospinasterol mixture and are shown in Table 1. The highest percent DPPH inhibition (49.46%) was obtained with a concentration of 50 µg mL^−1^ of the spinasterol and 22,23-dihydrospinasterol mixture, followed by 36.61%, 31.52%, and 19.98% inhibition with concentrations of 12.5, 3.0, and 0.78 µg mL^−1^_,_ respectively.

**Table 1:** Antioxidant activity of a mixture of spinasterol and 22,23-dihydrospinasterol.

Concentration (µg mL^−1^)	DPPH inhibition (%)
0.78	19.98 ± 1.66^a^
3	31.52 ± 0.94^b^
12.5	36.61 ± 0.79^c^
50	49.76 ± 0.12^d^
**Statistics**	
S.S	2270.36
M.S	756.79
D.F	3
F	11,140.69
*P*	0.000

Values are means of five replicates ± standard error. Different letters indicate significantly different values at the *P*=*0.05* level (Duncan’s multiple range test, DMRT).

### Antifungal activity

Table 2 presents data on the fungicidal activity of spinasterol and 22,23-dihydrospinasterol, as well as two synthetic chemicals, boscalid and carbendazim, which served as positive controls. The EC_50_ value for spinasterol and 22,23-dihydrospinasterol against *R. solani* was 129.56 µg mL^−1^, demonstrating the activity of the mixture against this fungus. Its activity against *M. grisea* was moderate, with an EC_50_ value of 206.09 µg mL^−1^, but it provided negligible control of *P. infestans*, with an EC_50_ value of 1093 µg mL^−1^. Boscalid was highly effective against *R. solani* and *P. infestans*, with EC_50_ values of 1.64 and 1.62 µg mL^−1^, but it was not effective against *M. grisea*, for which its EC_50_ value was 868 µg mL^−1^. By contrast, carbendazim showed excellent results against *M. grisea* and *R. solani*, with EC_50_ values < 0.78 µg mL^−1^, but it was ineffective against *P. infestans*, with an EC_50_ value of 8721.1 µg mL^−1^.

**Table 2:** Antifungal activity of spinasterol and 22,23-dihydrospinasterol against *Magnaporthe grisea*, *Rhizoctonia solani*, and *Phytophthora infestans.*

Compound name	Conc. µg mL^−1^	Inhibition ratio (%)			EC_50_^a^		
		*M. grisea*	*R. solani*	*P. infestans*	*M. grisea*	*R. solani*	*P. infestans*
Spinasterol and 22,23-dihydrospinasterol	0.78	0.088	0.016	0.158	206.09	129.56	1093.1
	3.12	0.115	0.073	0.195			
	12.5	0.218	0.208	0.247			
	50	0.373	0.336	0.341			
Boscalid	0.78	0.055	0.153	0.333	868.02	1.64	1.62
	3.12	0.139	0.913	0.616			
	12.5	0.212	0.964	0.966			
	50	0.255	0.964	0.994			
Carbendazim	0.78	0.955	0.653	0.118	< 0.78	< 0.75	8721.1
	3.12	0.997	0.879	0.187			
	12.5	1.000	1.000	0.141			
	50	1.000	1.000	0.170			

^a^EC_50_, half maximal effective concentration.

### Insecticidal activity

The data presented in Table 3 show the aphicidal activity of spinasterol and 22,23-dihydrospinasterol against the green peach aphid, *M. persicae*. In a residual assay in which adult aphids fed on individual, treated cabbage leaves in petri dishes, the highest mortality was observed after 72 h of exposure, with an LC_50_ of 42.46 µg mL^−1^, followed by 54.86 µg mL^−1^ at 48 h and 180.9 µg mL^−1^ at 24 h. Likewise, the highest mortality in a greenhouse assay was also recorded after 72 h, with an LC_50_ of 32.71 µg mL^−1^, followed by 42.46 µg mL^−1^ at 48 h and 173.8 µg mL^−1^ at 24 h. Mortality was therefore higher in the greenhouse assay than in the residual assay. The results presented in Table 4 show that after a prolonged exposure period of 72 h at a 50 µg mL^−1^ concentration, 63.3% mortality was observed in the greenhouse and 56.7% mortality in the residual assay. Higher mortality in the greenhouse than in the residual assay was also observed at 48 h (56.7% vs. 50%) and at 24 h (30% vs. 26.7%) at the 50 µg mL^−1^ concentration. Imidacloprid was used as the positive control at a rate of 0.0025 mL mL^−1^ water. It produced the highest rates of mortality after 72 h of exposure: 98.33% in the greenhouse assay and 96.67% in the residual assay. Likewise, Imidacloprid also produced significant mortality after 48 h of exposure: 91.67% in the greenhouse assay and 88.33% in the residual assay.

**Table 3:** Probit analysis of spinasterol and 22,23-dihydrospinasterol against *Myzus persicae*.

Bioassay	Time (h)	LC_50_ (µg mL^−1^)	95% F.L		Slope ± SE	χ^2^
			Lower	Upper		
Greenhouse	24	173.8	59.77	6796	1.01 ± 0.31	1.04
	48	42.46	25.11	107.2	1.38 ± 0.29	1.99
	72	32.71	19.40	73.57	1.47 ± 0.38	2.18
Residual	24	180.9	65.58	9889	1.17 ± 0.38	0.56
	48	54.86	31.38	166.2	1.42 ± 0.32	1.28
	72	42.46	25.11	107.1	1.38 ± 0.29	1.99

*F.L.* Fiducial limits, *χ*^*2*^ chi-squared, *LC*_*50*_ lethal concentration.

**Table 4:** Insecticidal activity of spinasterol and 22,23-dihydrospinasterol against *Myzus persicae*.

Conc. (µg mL^−1^)	Mean mortality (%)					
	24 h		48 h		72 h	
	Residual	Greenhouse	Residual	Greenhouse	Residual	Greenhouse
0.78	0.00 ± 0.00^d^	0.00 ± 0.00^b^	0.00 ± 0.00^c^	0.00 ± 0.00^c^	0.00 ± 0.00^d^	0.00 ± 0.00^e^
3.12	3.33 ± 5.77^c^	6.67 ± 5.77^b^	6.67 ± 5.77^bc^	10.0 ± 10.0^bc^	10.0 ± 0.00^bc^	13.3 ± 5.77^c^
12.5	6.67 ± 5.77^b^	10.0 ± 0.00^b^	20.0 ± 17.3^b^	16.7 ± 5.77^c^	16.7 ± 5.77^b^	23.3 ± 5.77^b^
50	26.7 ± 5.77^b^	30.0 ± 10.0^b^	50.0 ± 10.0^a^	56.7 ± 5.77^b^	56.7 ± 5.77^a^	63.3 ± 5.77^b^
CK	0.00 ± 0.00^d^	0.00 ± 0.00^c^	0.00 ± 0.00^d^	0.00 ± 0.00^d^	3.33 ± 5.77^ cd^	3.33 ± 5.77^d^
+ control	81.67 ± 1.29^a^	86.66 ± 1.29^a^	88.33 ± 1.29^a^	91.67 ± 2.58^a^	96.67 ± 2.58^a^	98.33 ± 3.87^a^
**Statistics**						
S.S	1493	1826	5306	6600	6293	7826
df	4	4	4	4	4	4
M.S	373	456	1326	1650	1573	1956
*F*	18.6***	17.1***	15.3***	49.5***	78.6***	73.4***

Data are presented as mean ± standard deviation, and different letters indicate statistically significant differences at *P* < 0.05 (DMRT). *S.S.* sum of squares, *df* degrees of freedom, *M.S.* mean square, *F* *F*-test statistic, *CK* check. ***P < 0.001.

### Correlation of antioxidant activity with antifungal and insecticidal activities

Pearson’s correlation coefficients regarding the antioxidant activity of spinasterol and 22,23-dihydrospinasterol showed positive relationships at concentrations of 3.12 µg mL^−1^ and 0.78 µg mL^−1^, which indicated that an increase in concentration of spinasterol and 22,23-dihydrospinasterol resulted in an increase in other values and showed significant (*P* < 0.05) results with antioxidant activities. Moreover, the antifungal activity towards *M. grisea* (B), *R. solani* (C), and *Phytophthora* (D), the insecticidal activity in the residual assay (E), and the insecticidal activity in the greenhouse assay (F) showed strong positive, significant (*P* < 0.01) relationships, as presented in Table 5.

**Table 5:** Correlation of the antioxidant activity of spinasterol and 22,23-dihydrospinasterol versus antifungal and insecticidal activities.

Concentration (µg mL^−1^)	0.78	3.12	12.5	50
**(A)**				
0.78	1			
3.12	0.84	1		
12.5	− 0.48	− 0.87	1	
50	− 0.86	− 1.00*	0.86	1
**(B)**				
0.78	1			
3.12	0.97**	1		
12.5	0.94**	0.99**	1	
50	0.95**	0.99**	0.99**	1
**(C)**				
0.78	1			
3.12	0.99**	1		
12.5	0.98**	0.99**	1	
50	0.95**	0.96**	0.98**	1
**(D)**				
0.78	1			
3.12	0.71**	1		
12.5	0.75**	0.99**	1	
50	0.73**	0.97**	0.99**	1
**(E)**				
0.78	1			
3.12	0.69**	1		
12.5	0.58*	0.92**	1	
50	0.54*	0.93**	0.95**	1
**(F)**				
0.78	1			
3.12	0.63*	1		
12.5	0.60*	0.82**	1	
50	0.70**	0.73**	0.92**	1

A, antioxidant activity. B, antifungal activity against *M. grisea*. C, antifungal activity against *R. solani*. D, antifungal activity against *Phytophthora*. E, insecticidal activity in residual assay; F, insecticidal activity in greenhouse assay. *P < 0.05, **P < 0.01, significant Pearson’s correlation coefficients.

## Discussion

Medicinal plants are highly prized by humans for their wide variety of biologically active compounds that are used in the pharmaceutical and agricultural industries. These products show substantial potential as natural antioxidants and are also commonly used against various insects^25,26^.

*Citrullus colocynthis* is a valuable source of antioxidant potential; for example, a butanol extract from *C. colocynthis* fruit showed an IC_50_ value of 6 µg mL^−1^, and an aqueous extract of fruit had an IC_50_ value of 241.25 µg mL^−1^. Antioxidant properties of *C. colocynthis* leaf and root extracts have also been documented: 45.9%, 39.81%, and 36.65% DPPH inhibition from hexane, aqueous and ethanol leaf extracts, respectively, and 29.12%, 35.51%, and 33.83% inhibition from root extracts^12^. The results of Benariba et al.^27^ are also consistent with our findings; they reported inhibition of DPPH radicals by seed extracts of *C. colocynthis* with IC_50_ values of 500, 580, and 350 µg mL^−1^ for aqueous, hydro-methanolic, and ethyl acetate extracts, respectively. Analysis of *C. colocynthis* extracts has revealed the presence of various biochemical compounds, including tannins, terpenoids, flavonoids, and coumarins, that may be responsible for the pronounced antioxidant effects and other biological activities of this plant^28^. Initial phytochemical screening of *C. colocynthis* revealed the presence of numerous flavonoids and phenols and showed significant antioxidant activity: 88.8% from fruit extract with potential free radical scavenging consequences at a concentration of 2500 µg mL^−1^^4^. Phenolic and flavonoid contents were quantified in solvent extracts of *C. colocynthis* roots, leaves, and fruits to compare their antioxidant activities. The total phenolic and flavonoid contents in leaf extracts were 3.07–18.6 mg g^−1^ and 0.51–13.9 mg g^−1^ of dry sample, respectively, followed by root and fruit extracts. Leaf ethanol extracts showed the highest antioxidant activity and DPPH radical scavenging activity compared with root and fruit extracts^29^.

Chawech et al.^30^ reported the antibacterial activity of the isolated compounds cucurbaticin E and glucocucurbaticin E from *C. colocynthis* against *Bacillus cereus* and *Enterococcus faecalis*. The minimum inhibitory concentrations (MIC) were 0.625 and 1.25 mg mL^−1^, respectively. Moreover, all *C. colocynthis* extracts showed antibacterial activity against *Pseudomonas aeruginosa, Escherichia coli*, *Enterococcus faecalis*, and *Staphylococcus aureus*, as well as antifungal activity against four *Candidia* species, *Candida krusei*, *Candida glabrata*, *Candida parapsilosis*, and *Candida albicans*^31^.

Plant extracts and essential oils contain secondary metabolites, including toxic phenolic, steroid, and terpenoid compounds that are stored in plant cells and show bio-pesticidal properties against pathogens and insect pests. Moreover, these compounds are easily biodegradable, reducing their ability to cause severe damage to humans and the environment^32–34^. Review of the literature shows several examples of plant products used for plant protection against a broad spectrum of pathogenic fungi. For instance, thymol and carvacrol have antifungal activity against *Botrytis cinerea* and *Fusarium* spp., and results indicate that these compounds could be employed independently as fungicidal agents against various phytopathogenic fungi^35^. The *α-*cadinol and *t-*muurolol compounds isolated from *Calocedrus macrolepis* exhibit significant fungicidal activity against *Fusarium oxysporum* and *R. solani*^36^. Methanol extract from the rhizome of *Acorus gramineus* contains numerous chemical compounds, such as caryophyllene, a-asarone, methyl isoeugenol, and isoasarone safrole, that show antifungal activity. In particular, asaronaldehyde (2,4,5-trimethoxybenzaldehyde) enabled complete control of *Phytophthora infestans* in potatoes and tomatoes and 75% control of *R. solani*^37^. Our findings on the antifungal activity of triterpenoids (spinasterol and 22,23-dihydrospinasterol) are consistent with those of Quiroga et al.^38^, who showed that lactones, sesquiterpene, and triterpenes from *Schinus molle* fruits and leaves had antifungal potential against *Alternaria alternata, Penicillium cyclopium, Aspergillus niger, Aspergillus flavus*, *Microsporum griseum*, and *Penicillium italicum*. Similarly, the flavonoid 4′-methoxy-5,7-dihydroxyflavone 6-*C*-glucoside isolated from the stems and leaves of *Aquilegia vulgaris* showed antifungal activity against the mold *A. niger*^39^. The antimycotoxigenic and antifungal activity of alcohol and distilled water extracts of *C. colocynthis* were evaluated against *A. flavus* and *Aspergillus ochraceus*, and they showed excellent antifungal activity against *A. ochraceus* with good antiochratoxigenic activity in liquid medium, consistent with findings about the antifungal activity of the triterpenoids spinasterol and 22,23-dihydrospinasterol^40^.

Activities of camphor, pulegone, and verbenone isolated from *Myristica fragrans* were assessed against the German cockroach *Blattellea germanica*, and these compounds showed LC_50_ values of 0.07 mg cm^−1^, 0.06 mg cm^−1^, and 0.07 mg cm^−1^, respectively^41^. Similarly, other compounds such as carvecol, eugenol, *p*-cymene, isoeugenol, and thymol displayed anti-adulticidal potential against *B. germanica* at a rate of 1 mg adult^−1^^42^. Likewise, spinasterol and 22,23-dihydrospinasterol exhibited medicinal and cytotoxic properties; these compounds were characterized in *Bougainvillea spectabilis* and exhibited marked inhibition of the enzyme xanthine oxidase, with an IC_50_ value of 39.21 µM^16^. Our results on the toxicity of spinasterol and 22,23-dihydrospinasterol showed that they exhibited insecticidal activity and caused significant mortality of *M. persicae*. Similar outcomes were described by Torkey et al.^43^, who reported that 2-*O*-*β*-d-glucapyranosyl cucurbitacin E isolated from *C. colocynthis* showed toxicity against *Aphis craccivora*, causing substantial mortality with an LC_50_ of 11,003 ppm. Moreover, 9-oxo-10,11-dehydroageraphorone isolated from *Eupatorium adenophorum* caused 73.33% mortality of *Pseudoregma bambucicola* at 2 mg mL^−1^ with a 6-h exposure. Moreover, 100% control of this pest was recorded at a similar concentration after one month of exposure in a field experiment^44^.

Contact toxicity of the new botanical insecticide Dayabon (SL 10%) was evaluated for different life stages of *M. persicae.* Its estimated LC_50_ values for first, second, third, and fourth instar nymphs and adults were 3254, 3387, 4194, 3839, and 3508 ppm, respectively, and it did not leave residues^45^. *Solanum incanum* fruit sap extract at different concentrations showed some level of insecticidal and deterrent activity against green peach aphid^46^. The insecticidal and deterrent activity of *Solanum incanum* may be attributed to the presence of saponins, which alter feeding behavior and molting, causing death at different developmental stages^31–33^.

The efficacy of *Xanthium strumarium*, *Tanacecetum parthenium*, and *Hypericum calycinum* extracts towards *M. persicae* was assessed; they produced nymphal mortality of 89%, 88%, and 57%, respectively, and adult mortality of 12%, 82%, and 88% at the same concentration^47^. Similarly^48^, the leaf extracts of several plants were evaluated against *M. persicae*, and *Ricinus communis* extract was most toxic to *M. persicae* (553 ppm), followed by extracts of *Robinia pseudoacacia* (1150 ppm for a 24-h exposure) and *Lantana camara* (6660 ppm). Another study^49^ reported that essential oil from *F. vulgare* caused significant mortality; this mortality was attributed to major compounds such as trans-anethole (67.9%) and fenchone (25.5%), with LC_50_ = 0.6 2.4 mL L^−1^ and LC_90_ = 2.4 mL L^−1^, and the oil was safe for non-target organisms. These results are consistent with our results on mortality of *M. persicae* following application of spinasterol and 22,23-dihydrospinasterol.

Our results also showed that the antioxidant activity of spinasterol and 22,23-dihydrospinasterol was significantly correlated with antifungal and insecticidal activity.

Although multiple studies have investigated the antioxidant, antimicrobial, antifungal, and insecticidal activities of plant extracts, essential oils, and isolated compounds, such activities have not previously been evaluated for spinasterol and 22,23-dihydrospinasterol. Thus, this research represents the first investigation of their antioxidant and antifungal properties and extends previous findings on their aphicidal activity against adult *M. persicae*.

## Materials and methods

### Collection of materials

Leaf samples of *C. colocynthis* (Cucurbitales: Cucurbitaceae), also known locally as *tumba*, were collected from a desert area of Punjab Province, Pakistan (29° 59′ 34″ N, 73° 15′ 13″ E) during 2019. The collected plant samples were identified as (Colocynthis) *C. colocynthis* by Dr. Dilbar Hussain Entomologist and Hafiz Naveed Ramzan Agronomist at the Entomological Research Institute, Ayub Agriculture Research Institute, Faisalabad, Pakistan. However, a voucher specimen of this material was not deposited because of the lack of an available herbarium. As this plant grows widely in vast, uncultivated desert regions and is partially used on a commercial basis, no permissions or licenses were required for sample collection.

Pure colonies of three pathogenic fungi, rice blast (*M. grisea*), sheath blight (*R. solani*), and *Phytophthora* (*P. infestans*), were obtained from Department of Pesticides Science, College of Plant Protection, Shenyang Agricultural University, Shenyang, China. The green peach aphids were collected from peach plants and were sustained on cabbage plants grown in a greenhouse at 20 ± 5 °C and 45 ± 5% relative humidity (RH) with a 16 h light/8 h dark photoperiod.

### Extraction, purification, and identification of biochemical compounds

Extraction, separation, purification, and identification of the purified compounds were performed by solvent/cold extraction, various chromatographic techniques, mass spectroscopy, and nuclear magnetic resonance (NMR) spectroscopy (^1^H-NMR and ^13^C-NMR), respectively, as previously documented by the author^11^ and are detailed in Supplementary File S1.

### Determination of radical scavenging activity using DPPH

We assessed the antioxidant activity of spinasterol and 22,23-dihydrospinasterol at various concentrations (0.78, 3.00, 12.5, and 50 µg ml^−1^) in Tween 20 (1% solution in distilled water) using the stable free radical molecule 1,1-diphenyl-2-picrylhydrazyl (DPPH) (C_18_H_13_N_5_O_6_), a dark-colored crystalline powder. In brief, 0.25 mL samples of various concentrations of the purified compounds prepared in methanol were added to 3.5 mL freshly prepared DPPH solution (0.002 g 50 mL^−1^ in HPLC grade methanol); the mixtures were shaken and incubated in darkness at 28 °C for 30 min. Subsequently, their absorbances were measured at 517 nm with a microplate reader (SpectraMax 190, Molecular Devices, made in China and designed in the USA), and percent inhibition of the prepared DPPH solution was calculated based on the decrease in absorbance using Eq. (1). A lower absorbance value indicated higher radical scavenging activity.

1 $$Inhibition \left(\%\right)=\frac{{A}_{blank}-{A}_{sample}}{{A}_{blank}}\times 100$$

where *A*_*blank*_ is the (absorbance of the control treatment) and *A*_*sample*_ is the (absorbance of the prepared sample).

### Determination of antifungal activity

The antifungal activity of spinasterol and 22,23-dihydrospinasterol against *M. grisea, R. solani*, and *P. infestans* was evaluated in vitro by radial growth tests on potato dextrose agar (PDA). The commercial synthetic fungicides boscalid and carbendazim were used as positive controls. The purified compound was dissolved in acetone and then mixed with PDA to obtain various concentrations (0.78, 3.0, 12.5, and 50 µg mL^−1^). The prepared PDA was transferred into 90-mm petri dishes (15 mL per dish) and inoculated with 5-mm pieces of *M. grisea, R. solani*, or *P. infestans*. The pieces of fungus were obtained by pressing at the corner of a mycelial colony already growing on PDA medium. After incubation for one week at 25 °C, the radius of mycelial growth was used to calculate the inhibition percentage of each chemical treatment relative to the 1% acetone control (CK). All treatments were replicated three times, and data were analyzed by standard methods.

### Determination of aphicidal activity

Aphicidal activity against the green peach aphid *M. persicae* was assessed in vitro (residual) and in vivo (greenhouse). For the residual assay, freshly cut cabbage leaves were dipped for 10 s in various concentrations of the tested compounds, dried, and placed in glass petri dishes. Next, 10 adult wingless aphids were transferred onto the leaves. The check (CK) was prepared using a 1% Tween 20 solution with no additional compounds, and all petri dishes were incubated at room temperature and 60% RH with a 16 h light/8 h dark photoperiod for 72 h. For the greenhouse assay, 10 adult wingless aphids were released on clean and healthy plants at the 5–7 true leaf stage. One hour after release, when the aphids had completely settled on the plant leaves, the plants were sprayed with various concentrations of compounds (2–3 sprays; 10 mL each) using a hand sprayer. Imidacloprid 25% WP (wettable powder) was used as the positive control at a rate of 0.0025 mL mL^−1^ of water, and control (CK) plants were sprayed with a 1% Tween 20 solution. Treated plants, positive controls, and CKs were placed in a greenhouse for 72 h.

Mortality data for the in vitro and in vivo experiments were collected after 24, 48, and 72 h of exposure by examining the aphids using a stereomicroscope. Individual aphids were considered to be dead if they made no response to needle stimulation.

### Correlation of antioxidant activity with antifungal and insecticidal activity

The correlations between antioxidant activity of spinasterol and 22,23-dihydrospinasterol and antifungal activity (rice blast, sheath blight, and *Phytophthora*) and insecticidal activity were calculated using IBM-SPSS statistics version 25.0 and assessed at the *P* < 0.05 significance level.

### Statistical analysis

Data were analyzed by analysis of variance (ANOVA), and differences among treatments were assessed using Duncan’s multiple range test (DMRT) at the *P* = 0.05 level using IBM-SPSS statistics version 25.0. Probability analysis was performed for the calculation of LC_50_ values using the EPA Probit analysis program version 1.5. Inhibition ratio and EC_50_ values were obtained using Log-Probit analysis.

### Statement of compliance

For experimental research, plants leaves were collected from wild habitat following institutional, national, and international guidelines and legislation. As the plant *Citrullus colocynthis* is wildly grown on vast uncultivated desert area and partially used on commercial basis so, no permissions or licenses was required for the collection of samples.

## Conclusions

Spinasterol and 22,23-dihydrospinasterol from *C. colocynthis* leaves showed moderate antioxidant activity, significant aphicidal activity against *M. persicae* in residual and greenhouse assays, and moderate antifungal activity against *M. grisea* and *R. solani*. Insect mortality was higher in the greenhouse assay than in the residual assay. The antioxidant activity of spinasterol and 22,23-dihydrospinasterol was strongly positively correlated with antifungal and insecticidal activity. Based on these findings, spinasterol and 22,23-dihydrospinasterol could be used for antioxidant, antifungal, and insecticidal purposes as an alternative to synthetic chemical agents. However, more research is needed on the isolation and characterization of other bioactive compounds and their evaluation as antioxidant, antifungal, and insecticidal agents.

## Supplementary Information

Supplementary Information.

## Data Availability

The data that support the findings of this study are available in the manuscript.

## Abbreviations

DPPH: 1,1-Diphenyl-2-picrylhydrazyl
EC_50_: Half maximal effective concentration
LC_50_: Lethal concentration
ABTS: 2,2′-Azino-bis-3-ethylbenzothiazoline-6-sulfonic acid
TEV: Tobacco etch virus
CMV: Cucumber mosaic virus
Conc.: Concentration
PDA: Potatoes dextrose agar
WP: Wettable powder
