# Supplementary material for: Antioxidant, antifungal, and aphicidal activity of the triterpenoids spinasterol and 22,23-dihydrospinasterol from leaves of Citrullus colocynthis L
Source: Sci Rep. 2022 Mar 22;12:4910. doi: 10.1038/s41598-022-08999-z (PMC8940894; doi:10.1038/s41598-022-08999-z)
Supplement: Supplementary file 1 — Supplementary Information. [file 41598_2022_8999_MOESM1_ESM.docx]

**Mass Spectrum and Elemental Analysis**

The C, H and N elemental analysis is helpful for the calculation of empirical formula. The mass spectrometer examination recorded the molecular ion peak (M) at *m*/*z* 414 and 412. These data suggested the expected molecular formula as a mixture (referred as D2 (N1) and D2 (2N) of C_29_H_50_O, C_29_H_48_O (Figure 1a-c). However, this compound is a mixture with the ratio of 5:4 calculated by the mass spectrum and nuclear magnetic resonance analysis.

| 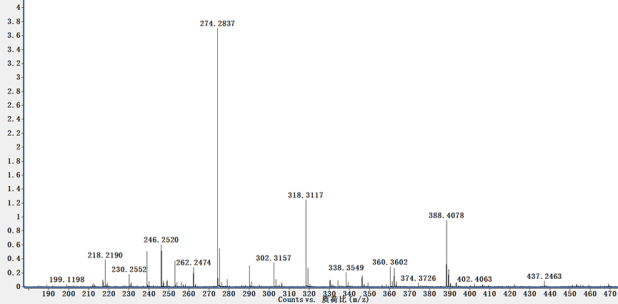 |  |
| --- | --- |
| (**a**)  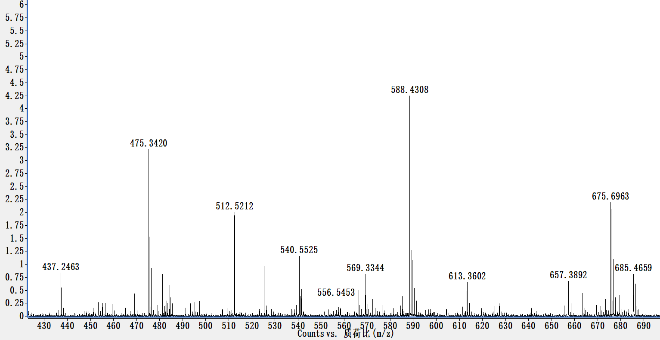  (**b**) |  |
| 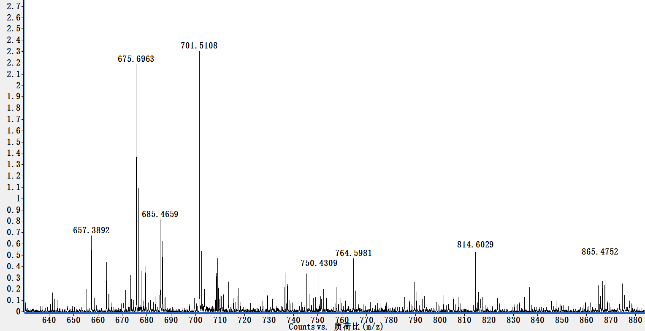 |  |
| (**c**) |  |

**Figure 1. a–c.** The full mass spectrum Spinasterol and 22, 23-dihydrospinasterol

**NMR Analysis (^1^H-NMR and ^13^C-NMR)**

The chemical structures of the purified compounds D2 (N) was characterized by nuclear magnetic resonance (NMR), Bruker BioSpin, Billerica MA for 1D (^1^H-NMR and ^13^C-NMR), mass spectrum and elemental analysis. All NMR experiments were carried out at room temperature.

In the ^1^H-NMR and ^13^C-NMR spectrum of the compound D_2_ (N 1) ^1^H-NMR (600 MHz, CDCl_3_) *δ* 5.16 (m, 1H, H-7), 3.60 (m, 1H, H-3) **allylic protons,** 0.93 (d, *J* = 7.0 Hz, 3H, -CH_3_), 0.85 (t, *J* = 6.8 Hz, 3H, -CH_3_), 0.82 (d, *J* = 6.9 Hz, 3H, -CH_3_), 0.81 (d, *J* = 6.9 Hz, 3H, -CH_3_), 0.80 (s, 3H, -CH_3_), 0.55 (s, 3H, -CH_3_). ^13^C-NMR (151 MHz, CDCl_3_) *δ* 11.8, 12.0, 13.0, 18.8, 19.0, 19.7, 21.5, 22.9, 23.0, 26.1, 27.9, 29.1, 29.6, 31.4, 33.8, 34.1, 36.5, 37.1,37.9, 39.5, 40.2, 43.3, 45.8, 49.4, 55.0, 56.0, 71.0, 117.3, 139.5, (Spinasterol, C_29_H_50_O; 414.0 g/Mol) (Figure 2a). However, ^1^H-NMR and ^13^C-NMR spectra of the compound D_2_ (N 2) ^1^H-NMR (600 MHz, CDCl_3_) *δ* 5.16 (overlap, 1H H-7), 5.16 (overlap, 1H, H-22), 5.03 (dd, *J* = 15.1, 8.8 Hz, 1H, H-23), 3.60 (m, 1H, H-3), ) the **allylic protons,** 1.03 (d, *J* = 7.0 Hz, 3H, -CH_3_), 0.85 (t, *J* = 6.8 Hz, 3H, -CH_3_), 0.82 (d, *J* = 6.9 Hz, 3H, -CH_3_), 0.81 (d, *J* = 6.9 Hz, 3H, -CH_3_), 0.80 (s, 3H, -CH_3_), 0.54 (s, 3H, -CH_3_). ^13^C-NMR (151 MHz, CDCl_3_) *δ* 11.9, 12.2, 13.0, 18.9, 21.0, 21.3, 21.5, 22.9, 25.3, 28.4, 29.6, 31.4, 31.8, 34.1, 37.1, 37.9, 39.4, 40.2, 40.8, 43.2, 49.4, 51.2, 55.0, 55.8, 71.0, 117.4, 129.4, 138.1, 139.5, (22,23-dihydrospinasterol, C_29_H_48_O; 412.0 g/Mol) (Figure 2b).


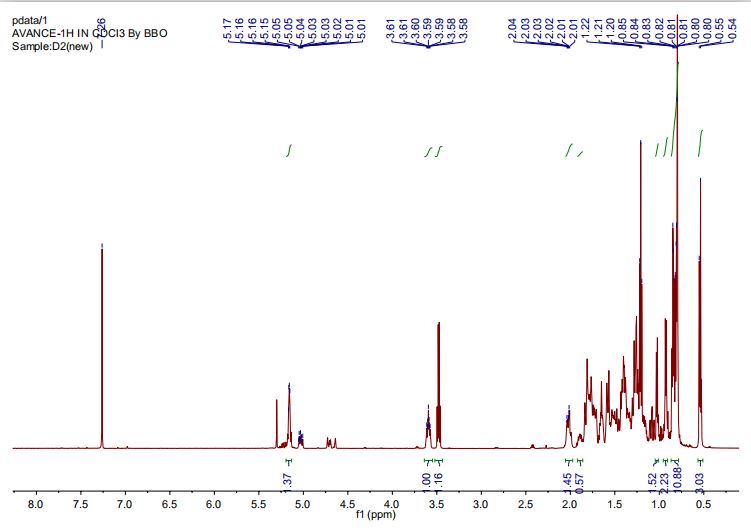


**(a)**


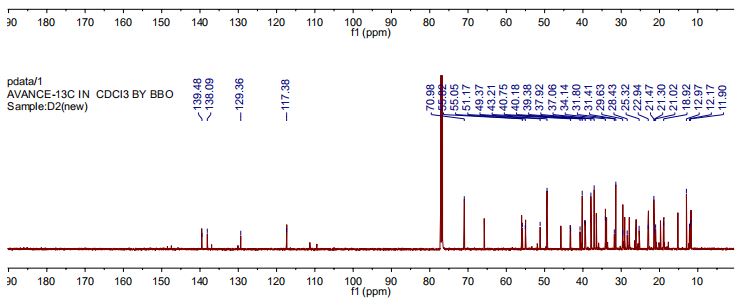


**(b)**

**Figure 2. a** ^1^H-NMR spectrum of the compound D2 (N); **b.** ^13^C-NMR spectrum of compound D2 (N)

**Structures Elucidation of the Compounds**

Chemical structures of the identified fractions of D2 (N) were characterized by ^1^H-NMR and ^13^C-NMR spectroscopy and their molecular formulae were predicted by the mass spectrum. Another purified compound was a little bit impure and due to this impurity, the ^1^H-NMR spectrum was overlapped and hence, mixture of two compounds was identified as Spinasterol and 22, 23-dihydrospinasterol from the fraction D2 (N) with ratio 5:4 in the mixture (Figure 3a and b).

.

|  |  |
| --- | --- |
| (**a**) | (**b**) |

**Figure 3.** Chemical structures of compound; (**a**). Spinasterol; (**b**) 22, 23-dihydrospinasterol
